# Timeliness of national notifiable diseases surveillance system in Korea: a cross-sectional study

**DOI:** 10.1186/1471-2458-9-93

**Published:** 2009-03-31

**Authors:** Hyo-Soon Yoo, Ok Park, Hye-Kyung Park, Eun-Gyu Lee, Eun-Kyeong Jeong, Jong-Koo Lee, Sung-Il Cho

**Affiliations:** 1 Division of Infectious Disease Surveillance, Korea Centers for Disease Control and Prevention, Seoul, Republic of Korea; 2 Division of Disease Control Policy, Ministry for Health, Welfare and Family Affairs, Seoul, Republic of Korea; 3 Korea Centers for Disease Control and Prevention, Seoul, Republic of Korea; 4 School of Public Health and Institute of Health and Environment, Seoul National University, Seoul, Republic of Korea

## Abstract

**Background:**

With the increase of international travels, infectious disease control is gaining a greater importance across regional borders. Adequate surveillance system function is crucial to prevent a global spread of infectious disease at the earliest stage. There have been limited reports on the characteristics of infectious disease surveillance in Asia. The authors studied the timeliness of the Korean National Notifiable Disease Surveillance System with regard to major notifiable diseases from 2001 to 2006.

**Methods:**

Six notifiable infectious diseases reported relatively frequently were included in this study. Five diseases were selected by the criteria of reported cases > 100 per year: typhoid fever, shigellosis, mumps, scrub typhus, and hemorrhagic fever with renal syndrome. In addition, dengue fever was also included to represent an emerging disease, despite its low number of cases. The diseases were compared for the proportion notified within the recommended time limits, median time lags, and for the cumulative distribution of time lags at each surveillance step between symptom onset and date of notification to the Korea Centers for Disease Control and Prevention (KCDC).

**Results:**

The proportion of cases reported in time was lower for disease groups with a recommended time limit of 1 day compared with 7 days (60%–70% vs. > 80%). The median time from disease onset to notification to KCDC ranged between 6 and 20 days. The median time from onset to registration at the local level ranged between 2 and 15 days. Distribution of time lags showed that main delays arose in the time from onset to diagnosis. There were variations in timeliness by disease categories and surveillance steps.

**Conclusion:**

Time from disease onset to diagnosis generally contributed most to the delay in reporting. It is needed to promote public education and to improve clinical guidelines. Rapid reporting by doctors should be encouraged, and unification of recommended reporting time limit can be helpful. Our study also demonstrates the utility of the overall assessment of time-lag distributions for disease-specific strategies to improve surveillance.

## Background

Effective public health services for the control and prevention of infectious diseases involve surveillance as a critical element [1]. The aim of infectious diseases surveillance is to initiate public health action in response to changes in the incidence of the disease [2]. The World Health Organization (WHO) has proposed two key functions of any surveillance system: early detection of potential public health threats, and monitoring of programs specific to single or multiple diseases [3]. In most countries, notifiable diseases are designated so that regular, frequent, and timely information can be generated by surveillance systems [4,5].

Timeliness is determined by the interval between any two steps within a surveillance system. Timeliness is a key measure of any surveillance system and should be assessed regularly [6-8]. It is a key element that is associated with the system's ability to take appropriate action on public health problems, based on the urgency and the type of responses needed [2]. Many studies have assessed the timeliness of surveillance systems by evaluating the time lag or bottleneck phenomenon of each surveillance step [2,8-11], comparing clinical and laboratory surveillance [12,13], and comparing electronic and conventional (paper-based) methods [10,14,15]. Recently, the WHO introduced guidelines for evaluating and monitoring surveillance systems. According to these guidelines, timeliness should be evaluated for specific surveillance steps for each disease and should be fully assessed from the time of infection to central reporting [3]. The conceptual framework provided by the WHO is useful to evaluate surveillance systems in many contexts.

In Korea, the Contagious Diseases Prevention Law was first enacted in year 1954 and laid the foundation of the National Notifiable Disease Surveillance System (NNDSS), by designating 20 notifiable infectious diseases for mandatory reporting. A major revision of this law in 2000 provided the current structure of the NNDSS, which collects individual patient information using an electronic reporting system. As of Aug, 2008, this system covers 50 infectious diseases.

NNDSS classifies infectious diseases into four groups: Group I for those requiring immediate control measures – 6 diseases (for example, cholera, plague, shigellosis); Group II for vaccine-preventable diseases – 9 diseases (for example, measles, mumps, rubella); Group III for diseases that need routine monitoring – 16 diseases (for example, tuberculosis, scrub typhus, malaria); and Group IV for emerging diseases in Korea – 19 diseases (for example, dengue fever, q fever, avian influenza infection in humans). The Korean NNDSS is organized at three levels: local, provincial, and central. At the local level, doctors report to the Public Health Center (PHC) when they diagnose a notifiable disease. Control measures, including epidemiological investigation and epidemic prevention, are conducted at this level by the PHC. At the provincial level, the PHC reports the cases to the Department of Health (DOH) of the province or metropolitan city. The DOH is obliged to control epidemics that may spread beyond a local district. The DOH reports the cases to the Korea Centers for Disease Control and Prevention (KCDC). The KCDC carries out control for multi-provincial outbreaks and assesses disease trends for monitoring and planned government action.

The magnitude and distribution of infectious diseases varies by region, and each country needs its own surveillance system and strategies for its particular situation. Although many studies of surveillance systems have been carried out to date, those in Asia have been rare. The new electronic data collection system of the Korean NNDSS provides a good opportunity to assess surveillance function. Therefore, our aim was to identify the timeliness of notifiable infectious disease surveillance in Korea.

## Methods

### Data collection

We used an anonymized database derived from NNDSS collected by the KCDC from 2001 to 2006. Five diseases were selected for this study that were reported in more than 100 cases every year for 2001–2006; typhoid fever, shigellosis, mumps, scrub typhus, and hemorrhagic fever with renal syndrome (HFRS). Reported in fewer numbers, dengue fever was also included to compare its differences with other disease groups. Despite having reports of more than 100 cases every year, tuberculosis and malaria were not selected because of insufficient information. Tuberculosis had been traditionally managed at the PHC by a different system, which did not include complete data on dates after physicians' notification step. Malaria cases included approximately 20% of the reports from military personnels covered by a separate reporting system within the military, and the information was not comparable for the current analysis. The six selected diseases represented four disease groups: Group I (typhoid fever and shigellosis), Group II (mumps), Group III (scrub typhus, HFRS), and Group IV (dengue fever).

### Definition of time lags

**Figure 1:**
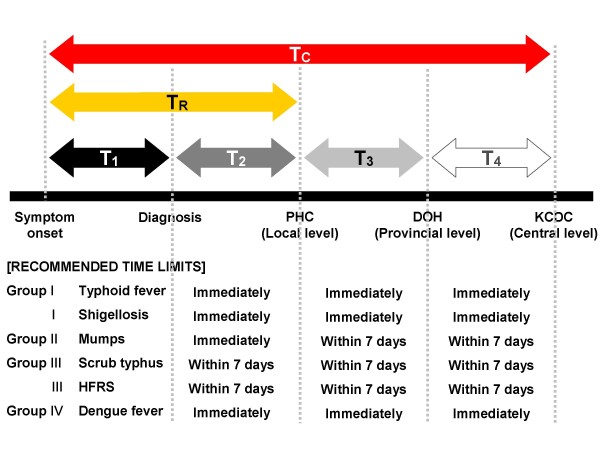
**Time points for NNDSS and recommended time limits for six selected diseases**. DOH, Department of Health (at the provincial level); KCDC, Korea Centers for Disease Control and Prevention (at the central level); PHC, Public Health Centers (at the local level).

Time points recorded in the surveillance data include dates of onset, diagnosis, doctor's notification to the PHC, PHC reporting to the DOH, and DOH reporting to the KCDC. Using these dates, we defined the time lags in days for each interval between the steps as T_1 _to T_4_, respectively. Additionally, the time to registration (T_R_) was defined as the delay from symptom onset to the doctor's notification to the local PHC. This time lag indicates the delay until the first recognition by the public health system that enables control action to be initiated. The time to central appraisal (T_C_) was defined as the delay from symptom onset to the notification received at the central level (KCDC). Central appraisal is a critical step that involves epidemiological verification of the reported data, statistical analysis, and interpretation. This step provides the basis for any central public health action nationwide. The recommended limits for each time lag are specified by the Contagious Diseases Prevention Law, according to the disease groups (Figure 1).

### Statistical Analysis

We analyzed the timeliness between the surveillance steps by calculating: (i) the proportion of cases reported within the recommended time limits; (ii) median time lags between the steps for each disease; and (iii) the cumulative distribution of time lags for diseases at each step. For time limits recommended as "immediately," an operational definition of 1 day was used for the timeliness criterion. Chi-square tests were performed to compare proportions of cases reported by the given time limit. Two-sample Kolmogorov-Smirnov (K-S) tests were used to compare cumulative distributions of time lags between diseases [16]. Analyses were performed using SAS version 9 (SAS Institute, Cary, NC) and Microsoft Office Excel 2003.

## Results

### Proportion of cases reported in time

For the six notifiable diseases examined, a total of 40,760 records were identified (Table 1). Scrub typhus comprised the largest number of cases (57.9%), followed by mumps (23.5%) and shigellosis (9.5%). The proportion of cases reported within the recommended time limit was calculated for T_2 _to T_4_. Generally, the time limit of 1 day was associated with a low proportion of cases reported in time, mostly ranging between 60% and 70%, compared to the 7-day limit, which had > 80% reporting in time. However, there was variation among the diseases for specific time lags. For example, doctors' timely reporting to the PHC was highest for shigellosis (85.3%) and lowest for dengue fever (60.2%) (p < 0.0001), although the limits were both 1 day. When the intervals of T_2 _to T_4 _were combined, the diseases in Group I and IV (diseases of immediate concern) had even lower proportions reported in time compared to the other groups (p < 0.0001). Approximately 35% of dengue fever cases were reported in time to the KCDC, whereas ≥ 90% of the diseases in Group II and III were reported in time. Comparison of proportions reported in time may not be appropriate between the disease groups with different recommended limits. Therefore, actual distribution of time lags was used for detailed comparison by disease groups.

**Table 1:** Number of notified cases and proportion (%) notified within the recommended time limits of law

Disease	Total no. of notifications	Proportion (%) notified within the recommended time limit^a^			
		
		T_2_	T_3_	T_4_	T_2 _+ T_3 _+ T_4_^b^
Group I		*(1 day)*	*(1 day)*	*(1 day)*	*(3 days)*
Typhoid fever	1,346	70.7	64.7	69.9	49.8
Shigellosis	3,877	85.3	63.7	64.7	53.3
Group II		*(1 day)*	*(7 days)*	*(7 days)*	*(15 days)*
Mumps	9,557	68.8	89.5	96.6	89.7
Group III		*(7 days)*	*(7 days)*	*(7 days)*	*(21 days)*
Scrub typhus	23,585	84.7	84.1	97.9	91.2
HFRS	2,295	84.0	86.1	96.6	90.6
Group IV		*(1 day)*	*(1 day)*	*(1 day)*	*(3 days)*
Dengue fever	100	60.2	49.0	83.0	34.7

HFRS, Hemorrhagic fever with renal syndrome.

^a^The recommended time limit for each step for a specific disease group is presented in parentheses. A recommended time limit of "immediately" was operationally defined as "within 1 day." See Figure 1 for definitions of T_2_, T_3_, and T_4_.

^b^T_2 _+ T_3 _+ T_4 _indicates the sum of the recommended time limits from diagnosis to notification to the Korea Centers for Disease and Control and prevention.

### Median time lag

**Figure 2:**
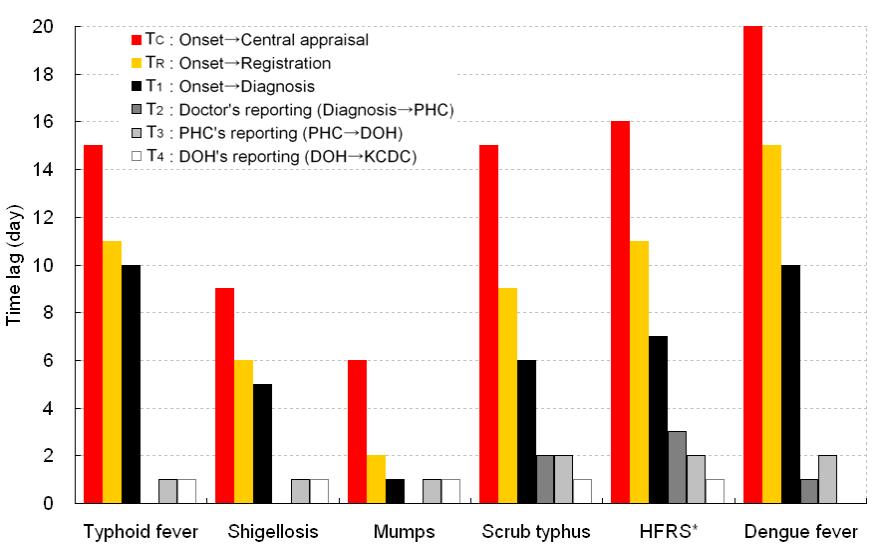
**Median time lags between the time points of surveillance**. DOH, Department of Health (at the provincial level); HFRS, Hemorrhagic fever with renal syndrome; KCDC, Korea Centers for Disease Control and Prevention (at the central level); PHC, Public Health Centers (at the local level). See Figure 1 for definitions of T_2_, T_3_, and T_4_.

Median time lags are shown in Figure 2. The T_C _and T_R _were shortest for mumps (6 and 2 days, respectively), followed by shigellosis (9 and 6 days, respectively). It is noteworthy that most time lags arose from a delay in diagnosis, especially for typhoid fever (T_1_, 10 days), dengue fever (T_1_, 10 days), and shigellosis (T_1_, 5 days). In contrast, T_1 _was just 1 day for mumps. Lags in the notification steps (T_2 _to T_4_) were generally longer for scrub typhus, and HFRS. Dengue fever showed longer delays in T_1 _and T_3_.

### Cumulative distribution of time lags

**Figure 3:**
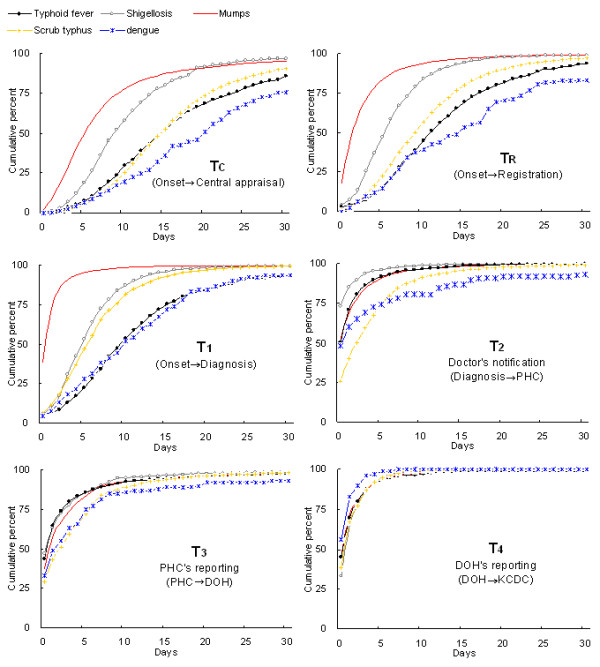
**Cumulative distribution of time lags by disease**. DOH, Department of Health (at the provincial level); KCDC, Korea Centers for Disease Control and Prevention (at the central level); PHC, Public Health Centers (at the local level).

Figure 3 compares the distribution of respective time lags among the diseases. Time lags for HFRS are not shown because their patterns were very similar to those of scrub typhus. The graph not only provides information on the median time lag by which 50% of the cases are reported for a specific disease, but also presents the overall shape of the time lag distribution. The higher curve indicates generally shorter time lags than those for lower curves. For example, T_C _curves show that at 10 days from the onset, more than 75% of mumps cases are notified to KCDC whereas approximately 50% of shigellosis and 25% of typhoid fever cases are notified, respectively. Comparing the cumulative distribution curves is much more informative than comparing fewer parameters such as median or mean, or proportions notified by given times. This is particularly true when the curves cross each other as shown by T_C _curves for typhoid and scrub typhus. Although the medians were the same with 15 days, the shapes of the distribution were significantly different from each other (p < 0.0001); Fewer typhoid fever cases were reported by 30 days than were scrub typhus cases.

Relatively large variation among diseases is apparent in both T_C _and T_R_; mumps (Group II) with the shortest and dengue fever (Group IV) with the longest time lags (p < 0.0001). This variation is largely due to a delay in diagnosis (T_1_) because these two diseases showed smaller difference in the other graphs. Shigellosis and typhoid fever, both in Group I, had relatively shorter time lags in the notification steps (T_2 _to T_4_). For these diseases, T_1 _appeared to be the critical step that contributed to the delay in T_C _and T_R_. However, there was a clear delay in doctors' reporting (T_2_) of typhoid fever compared to that of shigellosis. Doctors' notification of mumps to the PHC (T_2_) also appeared to involve a delay, with a curve almost overlapping with that for typhoid fever (p = 0.23), despite the fact that mumps had a very short time lag to diagnosis (T_1_). Scrub typhus (Group III) had a much shorter T_1 _than did typhoid fever. However, the difference became smaller for T_C _because it took a longer time for scrub typhus to be reported in the intermediate steps (T_2 _and T_3_). Dengue fever that represents Group IV showed the longest delay in T_C _and T_R_, primarily due to delays in both steps of diagnosis (T_1_) and doctor's report (T_2_).

## Discussion

We investigated the timeliness in different steps of surveillance for six relatively frequent notifiable infectious diseases in Korea. Typhoid fever and shigellosis had major delays in the time to diagnosis. In addition, typhoid fever had a longer time lag than did shigellosis in doctors' notification to the PHC. Mumps was relatively quickly diagnosed, but there was a delay in reporting by doctors. Scrub typhus, and HFRS had relatively greater time lags in reporting, partly because of the longer time limits that are recommended by Korean law.

Generally, we found shorter time lags compared to previous reports for these diseases. For shigellosis, we found a median T_C _of 9 days, whereas the T_C _ranged from 15 to 23 days in previous studies [2,8,10]. For typhoid fever, the median T_R _was 11 days, in comparison with approximately 22 days in a previous study in England [11].

Electronic reporting systems can be an important means to enhance timeliness, as previously suggested [10,15]. Our results showed generally shorter time lags than those of the previous studies using data from conventional systems. We used data collected by an electronic reporting system that was established in 2000, which would have contributed to the better timeliness compared to earlier data.

The level of timeliness varied according to the disease and surveillance step, with the total time lag from onset to central reporting ranging from 6 to 20 days. Previous studies have also described such differences according to diseases and surveillance steps [2,9,11,17]. For this reason, notification processes of infectious diseases cannot be assessed as a whole, but require analysis for separate clinical entities [9].

One of the main reasons for the variation in reporting among different diseases is the clinical characteristics of the diseases such as mode of onset and severity [15]. If the symptoms begin abruptly and progress severely, patients visit the doctor more quickly and diagnostic confirmation is faster. This probably explains the quicker reporting of mumps and shigellosis than the other diseases examined. In other studies, meningococcal infection and salmonellosis had shorter time lags than did tularemia or hepatitis A for the same reason [2,15]. Difficulty in diagnosis tends to delay the reporting of diseases by doctors because they need to balance the costs of false positives and delayed reporting. The necessity for time-consuming laboratory tests for diagnosis may also delay reporting.

Epidemiological characteristics such as incidence may affect timeliness in reporting, which can be facilitated by familiarity with the disease in the population. For example, measles is more common and thus more quickly reported than malaria in England [11]. Occurrence of an epidemic is likely to have similar effects. In fact, notifications of shigellosis in this study included 1,076 cases identified by KCDC as part of several different epidemics. Although in-depth analyses of these cases are beyond the scope of the current paper, the median T_C _and T_R _of the epidemic cases were shorter (8 and 5 days, respectively) compared to those of the other 2,801 non-epidemic cases (9 and 6 days, respectively).

The different patterns of time lags among the diseases examined suggest that there are common features among the disease groups, and strategies for better control should be developed specifically for each disease group. Such patterns by disease groups arise largely from the commonality of clinical and epidemiological nature, and the characteristics of surveillance policy as in Korea. Typhoid fever and shigellosis (Group I) had relatively long time lags to diagnosis, with a median of 10 and 5 days, respectively. It should be noted that these water- or food-borne diseases may result in secondary infection within this time period, causing a large epidemic. Previous studies of surveillance have tended to pay most attention to the time lags in reporting after physicians' diagnosis. However, the delay in diagnosis is also important because it may allow widespread transmission and lead to large epidemics.

Mumps (Group II) had the shortest time lag at every step. Its course is generally not severe and is self-limited. However, mumps is a common childhood viral disease that can induce outbreaks. Our results suggested some delay in physicians' notification of mumps to the PHC. This step needs attention and more investigation to improve the timeliness.

Scrub typhus and HFRS (Group III) seemed relatively limited in timeliness. These are among the most frequently reported infectious diseases in Korea. They are vector-borne diseases that mostly occur in autumn, especially in elderly people who are engaged in farming. Although the clinical course is not extremely severe and treatment is usually effective, early intervention is important to reduce morbidity and mortality, especially for vulnerable elderly groups. Measures such as intensive education and public health campaigns are needed for residents and physicians in high-risk areas and seasons. Moreover, the lenient 7-day time limit of reporting under the current law may cause unnecessary delay, and tightening of the limit should be considered.

Finally, dengue fever (Group IV) showed long delays in T_C _and T_R_. Dengue fever is not endemic in Korea and all cases are imported, as most diseases in Group IV. Because of its unfamiliarity, both diagnosis (T_1_) and doctor's report (T_2_) seem to be delayed. With recent increase in the number of imported cases, education for travellers and their physicians should be emphasized.

We used a conceptual framework for the surveillance system that involved two key parameters: T_C _and T_R_. Our approach was a simplification of the standard conceptual framework proposed by the WHO [3]. In our view, T_C _and T_R _capture the most critical aspects of surveillance function that enable responses to epidemics at central and local levels, respectively. These two key parameters were subdivided into the more detailed steps of T_1 _to T_4 _to identify the sources of delay and strategies to improve the system. It is also very important to examine the whole distribution of these time-lag parameters, rather than only a few statistics such as medians or means. The entire shape of the cumulative distribution may provide important information about the differences among diseases and specific surveillance steps.

Our study has some limitations. First, the disease-specific completeness of the Korean surveillance system ranges from 40% (HFRS) to 80% (shigellosis), which is comparable to those in other countries [1]. The unreported cases may have different characteristics from the reported cases. Therefore, had they been reported, their time lags might not have followed the same distributions. Although further investigation is needed, any improvement strategy for the current surveillance system would have to depend on the reported cases.

Second, laboratory confirmation is expected to vary according to the disease. The Korean surveillance system depends on the physicians' clinical diagnosis, regardless of laboratory confirmation. This is because the surveillance system aims at detecting all clinically relevant cases, so that transmission prior to laboratory confirmation is minimized. The diagnosis of mumps is typically made without laboratory tests. In contrast, the diagnosis of typhoid fever and shigellosis depends more on laboratory tests. Unfortunately, detailed information on the laboratory tests was not available for this study, and we were not able to stratify the analyses by the laboratory results. Further study is needed to assess how the laboratory confirmation process influences the timeliness of diagnosis or reporting by doctors.

## Conclusion

The infectious disease surveillance system in Korea generally functions well in terms of timeliness, although there are variations according to the disease and surveillance step. Three main approaches may improve the timeliness of the system overall. First, the main delays arise from time lags in the diagnosis, and these need to be improved by promoting public education and improving clinical guidelines. Second, more rapid reporting by doctors to PHCs should be encouraged. Third, the unification of recommended time limit as 1 day for all diseases will be helpful to provide simpler guidelines that can prevent needless time delay for reporting.

Methodologically, the assessment of the cumulative distribution of time lags at each step provides important information for evaluating the surveillance system and developing strategies for improvement.

## Abbreviations

WHO: the World Health Organization; NNDSS: the National Notifiable Disease Surveillance System; PHC: Public Health Center; DOH: Department of Health; KCDC: Korea Centers for Disease Control and Prevention; HFRS: Hemorrhagic Fever with Renal Syndrome.

## Competing interests

This work was partially supported by the Brain Korea 21 Project. The authors declare that they have no competing interests.

## Authors' contributions

HSY completed the analyses and led the writing. OP helped originate the study and interpret findings. HKP assisted in creating the analytic plan, and provided feedback on article drafts. EGL helped to interpret finding and review drafts of the article. EKJ was instrumental in the design, administration, and analysis. JKL reviewed draft and provided critical feedback on the draft. SIC supervised all stages of the study, including originating the study and developing the analytic plan, and writing the article. All authors read and approved the final manuscript.

## Pre-publication history

The pre-publication history for this paper can be accessed here:


